# Physicochemical, Pasting and Thermal Properties of the Starch of Three Varieties of Yam (*Dioscorea* spp.) as Potential Food Ingredients

**DOI:** 10.3390/polym18080943

**Published:** 2026-04-12

**Authors:** Mildreth Cordero-Herrera, José Benítez-Lobo, Claudia De Paula, Ricardo Andrade-Pizarro, Piedad Montero-Castillo, Diofanor Acevedo-Correa, Jhon Rodríguez-Meza, Alba Durango-Villadiego

**Affiliations:** 1 Master’s Program in Agri-Food Sciences, University of Córdoba, Córdoba 230002, Colombia; 2 Department of Food Engineering, University of Córdoba, Córdoba 230002, Colombia; cdepaula@correo.unicordoba.edu.co (C.D.P.); rdandrade@correo.unicordoba.edu.co (R.A.-P.); 3 School of Engineering, University of Cartagena, Cartagena de Indias 130000, Colombia; 4 Grupo de Investigación en Nutrición y Dietética, Universidad del Sinú Elías Bechara Zainum, Seccional Cartagena, Cartagena de Indias 130001, Colombia

**Keywords:** starch, yam, technofunctional properties, physicochemical properties

## Abstract

Yam starch accounts for 70–80% of its dry matter, and its physicochemical and technofunctional properties are crucial for its use in the food industry (gelling agent, thickener, and stabilizer). The objective of this study focuses on the physicochemical, pasting and thermal properties of starch extracted from the yam varieties *Dioscorea cayenensis*, *Dioscorea alata*, and *Dioscorea rotundata*. The proximal composition, amylose and amylopectin content, as well as their functional properties (absorption index, solubility, swelling, thermal and pasting behavior, morphology, and color) were analyzed. The results showed that the starch extraction yield varied between varieties, being highest in *D. cayenensis* with 14.14%. *D. alata* had the highest starch (82.24%) and amylose (34.69%) content, which gives it greater gel firmness and retrogradation potential, as well as the best techno-functional properties water absorption index (2.46 g/g), water solubility index (1.1%), and swelling power (2.54 g/g). *D. cayenensis* stands out for its high amylopectin content (69.62%) and brightness (96.89), reflecting greater starch whiteness. *D. rotundata* has an intermediate balance between amylose and amylopectin, which makes it versatile. The proximal composition and techno-functional properties of yam starch position it as a promising raw material for the food industry, especially in the manufacture of thickeners, gelling agents, and in bakery products, pasta and noodles.

## 1. Introduction

Yam (*Dioscorea* spp.) is a tuber belonging to the Dioscoreaceae family, a genus that includes more than 600 species [1]. It stands out as one of the fastest-growing crops in the economy, forming part of the diet of millions of people in Africa, Asia, Latin America, and the Caribbean [2]. It is cultivated by small and medium-sized farmers, constituting one of the main sources of food, income, and employment [3]. 98% of global production occurs in Africa, while America produces approximately 1.18%, with Colombia leading the region at 402,358.18 tons in 2022 [4]. The species of greatest economic importance in Colombia are *Dioscorea rotundata*, *Dioscorea cayenensis*, and *Dioscorea alata* [2,5].

Yam is of great importance and economic interest due to their nutritional value, being a good source of carbohydrates and minerals such as calcium, iron, and phosphorus, significant amounts of vitamins A and C; essential amino acids such as arginine, leucine, isoleucine, and valine [6], and an excellent source of fiber, sugars, and starch [7]. In addition, it stands out for its low-fat content and for containing saponins, particularly diosgenin, a chemical substance that has gained relevance in the industry, as it is used as a base for the production of steroids, which are essential in various pharmaceutical applications [3,8].

Yam is a good source of starch, which accounts for between 60 and 80% of the dry matter in this tuber [9]. Starch granules are made up of linear amylose and branched amylopectin, and amylopectin has a highly branched structure. These components contribute to the semi-crystalline nature of starch [10]. Generally, its composition has a ratio of approximately 20–30% amylose, and 70–80% amylopectin [11]. This composition varies depending on the plant from which it is extracted. In the case of the species *Dioscorea alata*, the amylose composition has been found to reach up to 30% [12], and in the varieties of *Dioscorea rotundata*, the concentration of amylose in the starch has been reported to range from 17.45% to 28.3% [13].

Starch stands out as a versatile raw material for improving the texture and consistency of foods, as it allows their physical properties to be adjusted according to the needs of the final product. Its functionality depends on the average molecular weight of amylose and amylopectin, as well as the molecular organization of these glucans within the granule [14]. In general, high levels of amylopectin provide elasticity and plasticity [15], while amylose promotes the formation of firm and transparent polymeric structures [16]. However, the optical and thermal properties of starch do not depend exclusively on the amylose/amylopectin ratio, as ions such as Ca^2+^, Mg^2+^, Na^+^, and K^+^ can modify transparency, gelatinization, and thermal stability through interactions that alter the molecular organization of the polymer [17,18]. In particular, these cations can form complexes with hydroxyl groups in starch, affect its crystallization, and modify the glass transition, thus influencing both the viscosity and thermal stability of the system [19,20].

In the food industry, starch serves multiple functions as a gelling agent, thickener, encapsulant, emulsion stabilizer, and film former [21,22,23]. Its widespread use is attributed to its physicochemical (gelatinization and retrogradation) and functional properties, such as solubility, swelling capacity, water absorption, syneresis, and rheological behavior of its pastes and gels [24], as well as its biodegradability, renewability, and abundance. Given that each botanical source has particular structures and behaviors, the characterization of starch from different varieties of yam is essential to identify specific applications, optimize transformation processes, and evaluate its potential as an alternative to traditional commercial starches.

The yield and techno-functional properties of starch vary considerably between species and varieties of yam, making it necessary to characterize them in order to improve both extraction efficiency and agro-industrial utilization [25]. Knowledge of attributes such as viscosity profile, solubility, syneresis, and retrogradation facilitates the design of physical, chemical, or enzymatic modifications for specific uses. Recent studies demonstrate, for example, the application of modified starch from *D. alata* in instant soups, as well as the potential of vegetable starches in bioplastics, edible films, and encapsulating matrices [26]. Overall, accurate characterization not only provides valuable scientific information, but also strengthens the industrial value of these yam varieties, promoting their development as a sustainable and strategic raw material [27].

The comprehensive evaluation of the physicochemical, thermal, and pasting properties of starch from different yam varieties, including *D. rotundata*, *D. cayenensis*, and *D. alata*, is of paramount importance for determining their potential industrial and food applications, as these properties are highly species and even cultivar specific. Furthermore, these properties are determined not solely by genetics but also by the specific agroecological conditions of the growing site, including soil characteristics and climatic factors [28,29]. The objective of this study was to determine the nutritional composition and physicochemical, pasting and thermal properties of starch extracted from yams of the varieties *Dioscorea cayenensis* (yellow), *Dioscorea alata* (Diamante), and *Dioscorea rotundata* (Espino) to establish its appropriate use in industry as a thickener, gelling agent, and stabilizer in various foods.

## 2. Materials and Methods

### 2.1. Raw Materials

Three varieties of yam were used: *Dioscorea cayenensis*, *Dioscorea alata*, and *Dioscorea rotundata*, freshly harvested and grown in the departments of Córdoba and Sucre, Colombia.

### 2.2. Extraction of Starch from Yams

The extraction of yam starch was carried out according to the methodology of Durango et al. [30], with some modifications. The yams were cut into pieces approximately 4 cm long and crushed in an industrial blender; the resulting suspension was sieved through a veil-type cloth and left to settle for approximately 24 h at 4 °C, due to the presence of mucilage that hindered the settling process. The decanted starch was washed four times with distilled water until it turned white. The wet starch was placed in a hot air tray dryer at 50 °C for 16 h, until it reached a moisture content of approximately 10–12%. It was then ground using a mortar and the powder obtained was sieved through a 200 μm mesh. The starch extraction yield was determined by calculating the ratio between the final weight of the dry starch obtained and the weight of the yam pulp used in the extraction, according to Equation (1) [31].

(1) $$Ys,\;\%=\frac{W_{b}}{W_{a}}\times 100$$

where

*Ys*, %: Starch yield in percentage;

*W_b_*: Weight of the dry starch;

*W_a_*: Weight of the yam pulp.

### 2.3. Physicochemical Characterization

#### 2.3.1. Proximal Composition

The proximate composition was determined by analyzing moisture (method 931.04), crude protein (method 970.22), crude fat (method 963.15), crude fiber (method 930.20), ash (method 972.15), and carbohydrates by difference, according to the official methods described by AOAC International [32]. Minerals were determined according to the methodology reported by Zhao et al. [33] with modifications, using flame atomic absorption spectrophotometry for calcium and magnesium, with wavelengths of 422.7 nm and 285.3 nm, respectively, and flame atomic emission spectrophotometry for sodium and potassium with wavelengths of 589 and 766.5 nm, respectively, and calculations were performed according to Equation (2).

(2) $$\%mineral=\frac{Absorbance\times Dilution\;factor\times Final\;volume}{Weight\;sample}$$

#### 2.3.2. Starch, Amylose, and Amylopectin Content

The starch content was determined according to the methodology described by Salgado Ordosgoitia et al., by enzymatic hydrolysis, and the concentration of reducing sugars using the DNS method [34]. The amylose content was determined using the iodine colorimetric method described by Salgado Ordosgoitia et al. [34], with some modifications. 100 mg of defatted starch was dispersed in 1 mL of 95% ethanol and 9 mL of 1 M NaOH, then heated in a boiling water bath for 10 min to ensure complete gelatinization. After cooling, an aliquot was mixed with distilled water and iodine reagent, and the absorbance was measured at 600 nm using a UV-Vis spectrophotometer (Evolution 60S, ThermoFisher Scientific, Shanghai, China). The amylopectin content was determined by the difference between the total starch content and the amylose content.

#### 2.3.3. Determination of Water Activity

Water activity (aw) was measured according to Buhler et al. [35], with some modifications, using a Waterlab water activity meter (Steroglass, Perugia, Italy). Approximately 3 g of the sample was used and measurements were taken at a controlled temperature of 25 °C, with an accuracy of ±0.05 °C, ensuring stable and consistent conditions throughout the procedure.

#### 2.3.4. pH Determination

To determine the pH, the potentiometric method 981.12 was used, in accordance with AOAC International [32].

#### 2.3.5. Vibration Analysis

Vibrational characterization of the starch was performed according to the methodology described by Paluch et al. [36], using an FTIR spectrometer (IS20, Thermo Nicolet Inc., Waltham, MA, USA). Each sample was placed on the diamond glass to completely cover its surface and then the pressure application knob was gently lowered onto the deposited substance until it was pressed against the glass. Finally, the spectral region from 500 to 4000 cm^−1^ was set.

#### 2.3.6. Morphological Characterization

The morphology and birefringence of the starch granules were determined according to the methodology described by Medina et al. [37], using a microscope (C-B10, Optika, Bergamo, Italy), with some modifications. The microphotographs were obtained under bright and polarized light fields at 40× magnification, using a digital camera (Optika B10, Bergamo, Italy). For this purpose, 10 mg of sample was weighed and diluted in 1 mL of deionized water. A 20 µL aliquot was taken from this solution and poured onto a microscope slide to obtain the microphotographs.

#### 2.3.7. Color Determination

The color of the starch was determined using the methodology described by Narvaez Gomez et al. [38], using a colorimeter (CR-20 brand: Konica Minolta, Sydney, NSW, Australia) and the CIELab scale with a standard illuminant D65 (daylight) and an observation angle of 10° to obtain the parameters: L* (black (0) to white (100)), a* (green (−128) to red (+127)), and b* (blue (−128) to yellow (+127)).

### 2.4. Techno-Functional Properties

#### Water Absorption Index (WAI), Water Solubility Index (WSI), and Swelling Power (SP)

The water absorption index was determined according to methodology by Anderson [39] with some modifications; 0.5 g of dry starch was weighed in a centrifuge tube and 12.5 mL of distilled water, heated to 80 °C, was added. The samples were placed in a water bath at 60 °C for 30 min, shaking the tubes with the suspension every 15 min. They were centrifuged at 2700 RPM for 15 min, the supernatant (soluble starch) was removed, and the weight of the centrifuge tube containing the gel (insoluble starch) was recorded. The water absorption index (WAI) was calculated according to Equation (3).

(3) $$WAI=\frac{Gel\;weight\;(g)}{Weight\;sample\;(g)}$$

For the water solubility index (WSI), the supernatant was placed in a Petri dish, which had been previously dried and weighed. The supernatant was dried in an oven at 70 °C for 12–14 h, recording the weight of the Petri dish with the soluble part, and the WSI was calculated according to Equation (4).

(4) $$WSI=\frac{Weight\;of\;solids\;in\;the\;supernatant\;(g)}{Weight\;sample\;(g)}$$

The swelling power (SP) was calculated according to Equation (5).

(5) $$SP=\frac{Gel\;weight(g)}{Weight\;sample\;(g)-soluble\;weight\;(g)}$$

### 2.5. Differential Scanning Calorimetry

The starch gelatinization parameters were determined using a differential scanning calorimeter (DSC250) (TA Instruments, New Castle, UK), with some modifications. To prepare the sample, a mixture of 20% starch on a dry basis and 80% deionized water was used. Both components were carefully placed in an aluminum sample holder. The sample holder was hermetically sealed using a specific press (TA Instruments, T zero press, New Castle, UK) and kept at room temperature for 12 h to standardize the humidity. The sample was heated from 40 to 100 °C at a rate of 10 °C per minute. The thermogram was calibrated using an empty sealed aluminum sample holder as a reference. The thermal transition of the starch was defined in terms of T_o_ (onset), T_p_ (peak), and T_f_ (end), as well as the change in enthalpy (ΔH), and the thermogram was recorded using TA Universal Analysis software version 4.1D (TA Instruments, New Castle, UK) [31].

### 2.6. Pasting Properties

The pasting properties of yam starch suspensions were determined using a rheometer (Anton Paar, MCR 302, Graz, Austria) with a concentric cylinder geometry and a rapid starch analyzer (SAA24-2D/2V, Anton Paar, Graz, Austria). A yam starch suspension (4% *w*/*v*) was prepared and treated with a heating–cooling cycle. The temperature profile used was as follows: holding at 50 °C for 1 min; a heating ramp from 50 to 95 °C at a rate of 6 °C/min; holding at 95 °C for 5 min; then cooling back to 50 °C at a rate of 6 °C/min, and finally holding at 50 °C for 2 min. Spindle speed was set at 160 rpm in this analysis. The parameters of the initial pasting temperature (PT), peak viscosity (PV), final viscosity (FV), breakdown viscosity (BV), and setback viscosity (SV) were obtained. Data were processed using RheoCompass 1.12 software (Anton Paar, version 1.12 Austria).

### 2.7. Statistical Analysis

All tests were performed in triplicate and the data were expressed as mean ± SD (standard deviation). A simple statistical analysis was performed, and the significant difference in the data was analyzed using analysis of variance (ANOVA) and Tukey’s test with a significance level of 5%, using the Statgraphics Centurion XIX statistical software package (Statgraphics, The Plains, VA, USA).

## 3. Results and Discussion

### 3.1. Starch Extraction Yield

Similar yields were found for the three varieties, *Dioscorea cayenensis* (14.14%), *Dioscorea alata* (11.31%), and *Dioscorea rotundata* (11.60%), when extracting starch from yams. These values are higher than those reported for some yam varieties such as *D. bulbifera* L. at 7.44% [40] but lower than that found for *D. rotundata* of 20.89% [41]. In general, the manual starch extraction method has a low yield, but it is considered a good source due to its high purity [40,42]. The low yield of starch extraction from yams using conventional aqueous methods is fundamentally attributable to the presence and physicochemical interference of mucilage. This substance produces a high-viscosity suspension when macerated with water, thereby physically hindering the separation and sedimentation of starch granules during processing. Consequently, a significant portion of the starch, particularly the smaller granules, remains suspended in the viscous slurry and is lost with the discarded wash water, thereby drastically reducing the overall extraction yield [28,43]. No significant differences (*p* < 0.05) in yield were identified among the three varieties, which may be due to the use of the same extraction method and harvest time, which conditions the dry matter content of the tuber [44].

### 3.2. Physicochemical Characterization

#### 3.2.1. Proximate Composition, Starch Content, Amylose, and Amylopectin

The proximal composition of yam starch from the varieties *Dioscorea cayenensis*, *Dioscorea alata*, and *Dioscorea rotundata* is shown in Table 1. The moisture values obtained range from 6.52 to 10.20%, which is within the moisture ranges reported for the manual extraction and drying method used, and are adequate to prevent microbial growth. The protein and ash composition is within the ranges reported for the *Dioscorea alata* and *Dioscorea rotundata* varieties [31,34,42,45]. In general, the values of the proximate composition are low due to the starch hydro-extraction process, which removes most of the water-soluble proteins, ash, and fiber, resulting in their low presence in starches. This behavior is due to the hydrophilic nature of these components, and the low-fat content indicates that the raw material is not a significant source of fat [44,46].

According to Tukey’s test, significant differences (*p* < 0.05) were found in ash and carbohydrate content between the three yam varieties, which can be attributed to genetic factors and the differential capacity for mineral and starch accumulation during tuber development, soil, and agroecological growing conditions [31,45]. The variation in ash content among different yam starch varieties can be attributed to a complex interplay of genetic factors, environmental growing conditions, and intrinsic tuber characteristics. The content of ash in starch is contingent on the efficiency of mineral uptake and accumulation during tuber development. Genetic distinctions between species and cultivars are of primary significance, as evidenced by substantial variations in ash content among *D. rotundata* genotypes (0.24 to 0.86%). Environmental factors, including soil composition, climate, and agricultural practices, interact with genetic potential to modify mineral accumulation. The presence of ash content measuring less than or equal to 0.20% is indicative of starch of a satisfactory quality [47,48]. However, no significant differences were detected in crude fiber, protein, and Aw content between the *D. cayenensis* and *D. alata* varieties, which may be due to the genotypic similarity between these varieties and their similar response to hydro-extraction treatment, which reduces the soluble fraction [44,49]. Likewise, the ether extract had a low content, which is consistent with the low lipid content reported for yams, as this tuber is not a significant source of fat [42,46].

The mineral content present in the starch of the three varieties of yam (Table 1) shows that the main elements detected were calcium, magnesium, sodium, and potassium. Potassium was the most prominent element, showing significant differences (*p* < 0.05) in the *D. alata* variety (0.34 mg/kg) compared to *D. rotundata* (0.17 mg/kg) and *D. cayenensis* (0.10 mg/kg). This behavior is consistent with what has been reported in tubers and roots, where potassium is the main cation involved in osmotic and metabolic regulation. The differences observed between varieties for this and other minerals are associated with genetic factors and growing conditions. No significant differences were found in magnesium content between *D. cayenensis* and *D. rotundata*, nor in sodium content between *D. cayenensis* and *D. alata*, suggesting a relatively homogeneous distribution of these minerals among these varieties. However, the values found are considerably lower than those reported in other tubers such as arrowroot (*Maranta arundinacea*), with sodium (52.6 mg/kg), potassium (4312.95 mg/kg), and calcium (382.67 mg/kg) contents [50]; and those described in white and yellow yam flour, where concentrations of magnesium (650–720 mg/kg), calcium (190–210 mg/kg), sodium (20 mg/kg), and potassium (30 mg/kg) are reported [51]. These differences can be attributed both to genetic and crop-specific factors and to the fact that the extracted starch contains a very small mineral fraction compared to the whole tuber, due to the removal of water-soluble compounds during the hydroextraction process [44].

The total starch content (see Table 1) was higher in the *D. alata* variety (82.24%), followed by *D. rotundata* (70.08%) and *D. cayenensis* (64.86%), indicating that the *D. alata* has greater purity in the starch extraction process, which is of interest for industrial purposes and for its agro-industrial value. Although the three varieties showed similar total carbohydrate contents, the differences in starch are explained by the internal distribution of these carbohydrates, since the overall value includes not only starch, but also simple sugars, fiber, and other polysaccharides [52]. In addition, vegetable tubers store glucose reserves in the form of starch composed of amylose and amylopectin, the proportion of which can vary between species and affects their physicochemical properties [53].

The amylose content, *D. alata* starch had the highest value at 34.69%, which was significantly higher than *D. rotundata* at 33.24% and *D. cayenensis* at 30.34%. These values are higher than those reported for yam starches, 25.87 to 27.89% and 30.63%, potato 20.0% and 24% [40,54]. A high amylose content is associated with a greater tendency toward retrogradation, the formation of firmer gels, and the potential for obtaining resistant starch, which makes this variety suitable for the development of functional foods or slow-release carbohydrate products [55]. On the other hand, a high amylopectin content leads to greater swelling capacity, better paste stability, and a lower tendency to retrogradation, which are desirable characteristics in food applications such as sauces, creams, or baked goods [34,45].

#### 3.2.2. Vibration Analysis of Yam Starches

**Figure 1:**
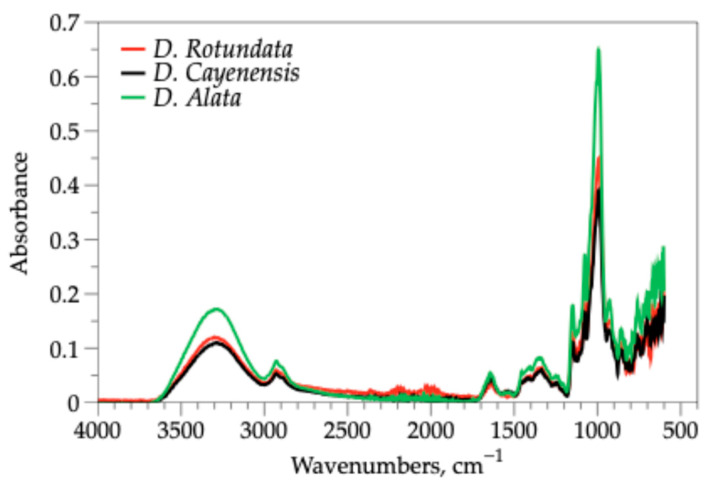
FTIR spectra of starch from three varieties of yam (*D. cayenensis*, *D. alata*, and *D. rotundata*).

The spectra obtained from Fourier Transform Infrared Spectroscopy (FTIR) of the native starch of the three varieties of yam are shown in Figure 1. Wide bands are observed in the range of 3600 to 3200 cm^−1^, similar to those found in native potato starch [56] and cassava starch. These bands are typical of polysaccharides, which are represented by the stretching of the OH bond, highlighting the presence of glucopyranose rings [57]. In addition, the stretching of the OH bond is observed as very strong and broad bands [58]. The shape of the band and the intensity corresponding to the OH groups varied mainly in the *D. alata* variety. This is related to differences in the local molecular environment of the water association forms present within the starch granule [59].

*D. cayenensis*, *D. alata*, and *D. rotundata* varieties of starch had wave numbers of 2928 and 2929 cm^−1^, which are similar to the values for cassava starch [60], falling within the range of 3000–2500 cm^−1^, a set of bands that are usually well defined and of moderate intensity, attributed to the symmetric and asymmetric stretching vibrations of the CH and CH_2_ groups present in the polysaccharide skeleton [61]. The bands 1642–1634 cm^−1^ represent a C-O bond bending associated with the OH group [62].

The three varieties of yam presented wave numbers between 1349 and 1337 cm^−1^, which involve vibrations of groups with local symmetry for CH_2_ and C-OH that commonly occur in carbohydrates [63]. In addition, wave numbers between 1149 and 1148 cm^−1^ were obtained, which are very close to the wave number 1157 found in cassava starch, potato [60,62] and millet [64], which represents a stretching of the C-O-C glycosidic bond. Additionally, wave numbers that can be attributed to C-O bond stretching have been found in the segment of the spectrum located in the region from 1200 to 800 cm^−1^ [62]. The main differences among *Dioscorea alata* and the other varieties are observed in the intensity of two regions of the spectrum: the OH vibration region, 3600–3200 cm^−1^, and the region of major adsorption bands, 1200–800 cm^−1^; the same difference has been reported for type A starches and types B and C.

The ratio of absorbances at 1047 cm^−1^ and 1022 cm^−1^ (R_1047_/_1022_) was calculated to evaluate the degree of short-range ordered structure in the starch granules, as this ratio is widely recognized as an indicator of crystalline order at the molecular level [65,66]. *D. alata* starch exhibited the highest R_1047_/_1022_ ratio, indicating greater short-range molecular order, which correlates with its higher amylose content and enhanced tendency to form rigid gels and resistant starch. In contrast, *D. cayenensis* starch showed a lower ratio, reflecting its higher amylopectin content and greater amorphous character, consistent with its superior paste stability and lower retrogradation tendency.

#### 3.2.3. Morphological Characterization of Yam Starches

**Figure 2:**
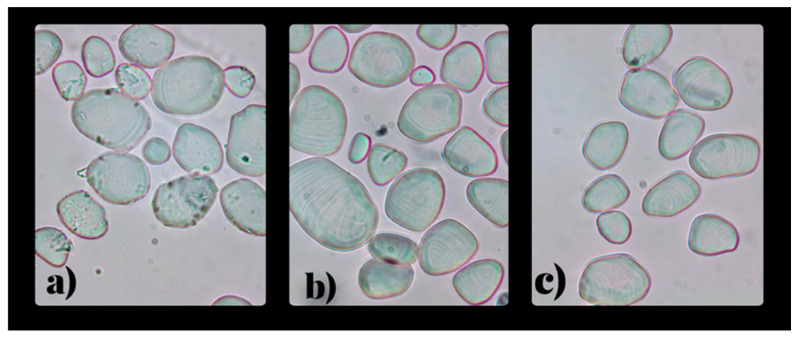
Bright field microscopy of starch: (**a**) *D. cayenensis* (**b**) *D. alata* (**c**) *D. rotundata*.

**Figure 3:**
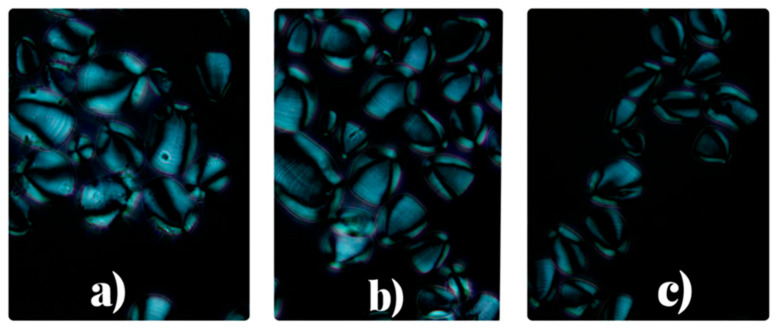
Polarized light microscopy of starch: (**a**) *D. cayenensis* (**b**) *D. alata* (**c**) *D. rotundata*.

Clear field and polarized light microscopy of starch granules from the varieties *Dioscorea cayenensis*, *Dioscorea alata*, and *Dioscorea rotundata* are shown in Figure 2 and Figure 3. The obtained granules had a defined size and shape, minimal presence of impurities, and a bimodal distribution (small and large granules) were observed. No damage, cracks, or pores were observed, confirming the efficiency of the extraction process and the preservation of intact structures. Likewise, an orderly arrangement characteristic of the semi-crystalline nature of starch was observed, with a helium point and growth in concentric rings [37,67]. Under polarized light, the granules showed characteristic birefringence or “Maltese cross,” associated with the alternation of crystalline and amorphous regions [68,69], confirming that the structural properties of the starch remained unchanged during extraction [67].

#### 3.2.4. Color Parameters of Yam Starches

The colorimetric parameters of the yam starch from the three varieties studied are reported in Table 2. The lightness (L*) of the starches from the yam varieties *D. cayenesis* (96.89), *D. alata* (96.01), and *D. rotundata* (96.56) are similar to that reported for kruda potato starch [70,71]. However, they are lower than that reported for cassava starch (99.28). A lightness close to 100 indicates a whiter starch.

For the color parameter a* of yam starch, the *D. cayenensis* and *D. alata* varieties have values between 0.21 and 0.46, respectively, indicating a slight tendency toward red, while the *D. rotundata* variety has a value of −0.67, indicating a slight tendency toward green. The b* color parameter of the yam starch of the three varieties showed values between 1.25 and 3.49, indicating a tendency toward yellow, and there are no significant differences (*p* < 0.05) between the *D. alata* and *D. rotundata* varieties. These parameters differ from those reported for cassava starch, with positive values of a* (0.02) and b* (0.89), indicating a tendency toward red and yellow, respectively [71,72].

Color is an important criterion for starch quality, especially for its use in various types of food products [73]. Starches that exhibit high brightness and reduced chroma values in the a* and b* parameters are particularly suitable for use in products that require a uniform and attractive color, such as ice cream, confectionery, sauces, and soups [31]. These properties also favor their use in the formulation of dairy and non-dairy beverages, where stability and clear appearance are quality attributes [74]. Similarly, their color neutrality and visual stability make them suitable for use in baby foods and dietetic products, where sensory acceptability is essential [75].

### 3.3. Functional Properties of Yam Starches

#### Water Absorption Index (WAI), Water Solubility Index (WSI), and Swelling Power (SP)

Table 3 shows the functional properties (WAI, WSI, and SP) of the starch from the three varieties of yam. For WAI, the values in yam starches range from 2.22 to 2.39 g/g, similar to the values reported for yam starches of various varieties, 2.32 to 2.39 g/g [45], and lower than those reported for cassava starches, 4.63 to 4.80 g/g [54], potato 5.83 g/g [54], and quinoa, 3.25 g/g [76]. For the varieties *Dioscorea cayenensis*, *Dioscorea alata*, and *Dioscorea rotundata*, significant differences (*p* < 0.05) were found in the water absorption index, which are related to the biological source, size, and shape of the granule.

The solubility indices at 60 °C obtained for yam starch range from 0.76 to 1.1%, which differ from those reported for yam starch of the *Dioscorea rotundata* variety (2.79%) [42] and the *Dioscorea alata* variety, which varies between 1.10–2.15% [45]. No significant differences (*p* < 0.05) were found between the yam starch of the *Dioscorea alata* and *Dioscorea rotundata* varieties, but there were differences with the *Dioscorea cayenensis* variety. The WSI is influenced by the composition and morphology of the starch granules. A higher amylose content, together with a smaller granule size, favors the penetration of water into the intermolecular spaces, as the linear chains dissociate more easily in hot water. In contrast, starches with a high amylopectin content, characterized by their highly branched structure, tend to retain greater structural integrity and, consequently, have lower solubility. These structural differences directly affect the solubilization capacity of starch in water and the stability of viscosity during gelatinization processes and technological applications [55,75,77,78]. The swelling power of the starch in the three varieties of yam ranges from 2.26 to 2.54 g/g, which are similar to those reported for other tubers such as makal, sweet potato, cassava, and sago [24] and the *Dioscorea bulbifera* L. variety of yam (2.72 to 3.15 g/g) [40]. The yam variety had a significant influence (*p* < 0.05) on SP, which may be due to the variation in amylopectin content, as its branched structure retains more water during gelatinization. This is a measure of the increase in non-solubilized starch mass as a result of water absorption by the hydroxyl groups of amylopectin polymers [42].

### 3.4. Differential Scanning Calorimetry of Yam Starches

**Figure 4:**
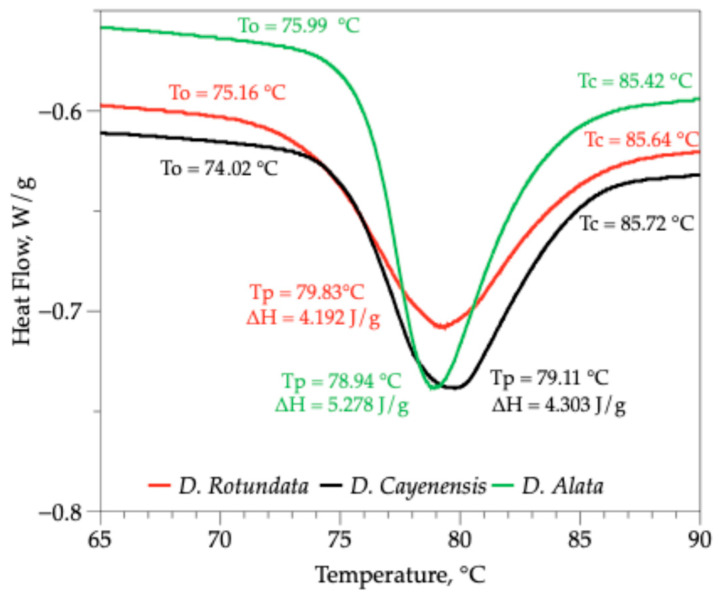
Differential Scanning Calorimetry (DSC) of starch from three varieties of yam (*D. rotundata*, *D. cayenensis* and *D. alata*).

The starches from the three varieties of yams showed characteristic endotherms throughout the gelatinization process of each starch, with no difference in their maximum gelatinization temperatures (Tp) (Table 4 and Figure 4). In addition, the results obtained show that the gelatinization temperatures of the starches evaluated were within narrow ranges, with initial temperature (To) values between 74.02 and 75.98 °C, peak temperatures (Tp) between 78.94 and 79.63 °C, and final temperatures (Tc) between 85.42 and 85.72 °C. The enthalpy of gelatinization (ΔH) varied more markedly, with values ranging from 4.19 to 5.28 J/g, indicating differences in the energy required for the transition. These values were very close to those reported for *Dioscorea hispida* starch, which has a To of 74.54 °C, Tp of 79.35 °C, Tc of 83.36 °C, and ΔH of 4.12 J/g [79]. However, they differ from those reported for *D. alata* (To = 69.62 °C, Tp = 74.81 °C, and ΔH = 12.15 J/g) [31], and native potato starches (To from 58.7 to 62.5 °C, Tp from 62.5 to 66.1 °C, Tc from 68.7 to 72.3 °C, and ΔH from 15.1 to 18.4 J/g) [80].

The significant differences in the melting enthalpy of yam starch are particularly related to the internal molecular organization of amylopectin, which contributes to a more orderly arrangement. However, the structure of starch granules due to the amylose–amylopectin ratio and degree of crystallinity is closely related to the differences observed in the temperatures of the various starches analyzed [81]. The three varieties of yam had Tc-To values between 11.70 and 9.43 °C (Table 4), lower than those reported for cassava starch (15.95 °C), Ratona Blanca potato (13.8 °C), and Andina potato (12.6 °C). These variations can be attributed to the heterogeneity in the size of the starch granules and the structural arrangement of amylose and amylopectin [82,83]. The higher ΔH value in *D. alata*, combined with its narrower gelatinization range, indicates that this starch presents a more homogeneous and perfect crystalline structure, likely with a more uniform distribution of amylopectin chains [84,85]. This suggests that starch from *D. alata* requires more energy (heat) to fully gelatinize, and may be more resistant to moderate thermal treatments, which is useful in products requiring retention of granule structure during processing.

### 3.5. Pasting Properties of Yam Starches

**Figure 5:**
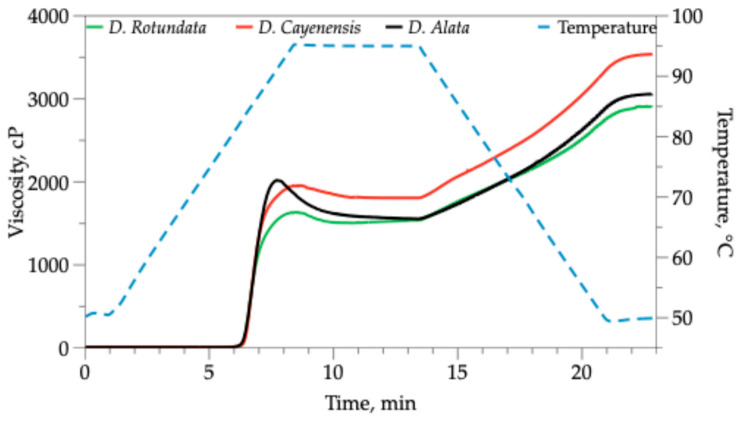
Pasting profiles of starch from three varieties of yam (*D. rotundata*, *D. cayenensis* and *D. alata*).

Pasting profiles of starch from three varieties of yam are shown in Figure 5 and pasting properties are summarized in Table 5. The yam starches had a lower peak viscosity compared to the final viscosity. When a starch suspension is subjected to heating, the granules undergo swelling and amylose leaching, resulting in a maximum viscosity level. Upon cooling, the solubilized linear amylose chains initiate the formation of double helices and subsequently aggregate into a three-dimensional gel network, thereby augmenting resistance to flow. In starches with elevated amylose content, the extent of this reassociation during the cooling phase can be so substantial that the resulting gel structure generates a final viscosity that exceeds the peak viscosity achieved during the swelling phase. This suggests that yam starches may be prone to retrogradation [78].

The pasting temperature ranged from 73.62 to 75.52 °C, values similar to those determined for the initial gelatinization temperature determined by thermal analysis (DSC) and consistent with those reported for cassava starches.

The ANOVA shows that there are significant differences in the viscosity peak between the starches of the three yam varieties, with the starches of the *D. alata* and *D. cayenensis* varieties having the highest values, which may be due to their larger granule size. The starches with higher viscosity peaks have a superior ability to thicken a food product during the cooking process, and can be applied in the preparation of sauces, gravies, and soups [79].

The final viscosity of the starches from the three varieties of yam showed significant differences (*p* ≤ 0.05), with the lowest being the starch from the *D. rotundata* variety and the highest from *D. cayenensis*, a high final viscosity indicates the starch has the potential to create a stable, thick gel when cooled, but also a greater susceptibility to hardening or aging during storage [80].

Yam starches exhibited low breakdown viscosity, particularly those from the *D. rotundata* and *D. cayenensis* varieties, which indicates greater resistance to heat and shear, an important condition for demanding industrial processing conditions. Setback viscosity was higher for starch *D. cayenensis*. This property is exhibited due to the recrystallization of amylose molecules in the gel, which is a measure of the retrogradation ability of starches [79,80].

## 4. Conclusions

The starches from the three varieties of yam (*D. cayenensis*, *D. alata*, and *D. rotundata*) have good extraction yields and low protein, fat, ash, and fiber content. Their techno-functional characteristics are promising, showing different behavior depending on their composition and molecular structure, which determines their functionality in the food industry. The three yam varieties exhibit distinct technofunctional properties directly linked to their molecular composition, highlighting the importance of varietal selection for specific food applications. The starch obtained from the *D. alata* stands out for its higher starch and amylose content, characteristics that favor the formation of firm gels, retrogradation, and the production of resistant starch, making it suitable for the formulation of functional foods, slow-release carbohydrate products, and the development of coatings and bioplastics with greater rigidity.

The starch from the *D. cayenensis* variety has a higher amylopectin content, a good swelling index, and paste stability. These properties make it more suitable for use in sauces, creams, desserts, dairy products, and baked goods, where viscosity, creamy texture, and less tendency to retrogradation are required. On the other hand, the starch obtained from *D. rotundata* has an intermediate balance between amylose and amylopectin, which makes it versatile as it can be used in sausages, soups, and bread mixes. These findings confirm that yam starch is a sustainable and low-cost alternative to conventional starches, with applications in functional foods, bakery products, breakfast cereals, pasta and noodles, and fat replacers and texture modifiers.

## Figures and Tables

**Table 1:** Proximate composition, starch content, amylose, and amylopectin content of yam starch.

Variety of Yam	*D. cayenensis*	*D. alata*	*D. rotundata*
Humidity (%)	10.20 ± 0.14 ^b^	8.60 ± 0.32 ^b^	6.52 ± 0.10 ^a^
Protein (%)	0.36 ± 0.01 ^a^	0.34 ± 0.02 ^a^	0.22 ± 0.01 ^b^
Fat (%)	0.25 ± 0.02 ^a^	0.29 ± 0.02 ^ab^	0.28 ± 0.02 ^b^
Ash (%)	0.04 ± 0.01 ^b^	0.12 ± 0.00 ^c^	0.02 ± 0.01 ^a^
Fiber (%)	0.24 ± 0.01 ^b^	0.24 ± 0.01 ^b^	0.21 ± 0.01 ^a^
Carbohydrates (%)	99.11 ± 0.02 ^b^	99.01 ± 0.04 ^a^	99.27 ± 0.11 ^c^
Calcium (mg/kg)	0.04 ± 0.00 ^a^	0.02 ± 0.00 ^b^	0.03 ± 0.00 ^c^
Magnesium (mg/kg)	0.02 ± 0.00 ^b^	0.01 ± 0.00 ^a^	0.02 ± 0.00 ^b^
Sodium (mg/kg)	0.01 ± 0.00 ^a^	0.01 ± 0.00 ^a^	0.02 ± 0.00 ^b^
Potassium (mg/kg)	0.10 ± 0.00 ^a^	0.34 ± 0.02 ^c^	0.17 ± 0.01 ^b^
Starch (%)	64.86 ± 4.91 ^a^	82.24 ± 0.49 ^b^	70.08 ± 8.64 ^a^
Amylose (%)	30.34 ± 0.77 ^a^	34.69 ± 1.42 ^b^	33.24 ± 1.64 ^b^
Amylopectin (%)	69.62 ± 0.85 ^c^	64.75 ± 0.81 ^a^	66.23 ± 1.45 ^b^
Aw	0.36 ± 0.01 ^b^	0.35 ± 0.00 ^b^	0.27 ± 0.00 ^a^

Results are reported on a dry basis. Different lowercase superscript letters in the same row indicate significant differences (*p* < 0.05).

**Table 2:** Color parameters of starch from three varieties of yam.

Parameter	*D. cayenensis*	*D. alata*	*D. rotundata*
L*	96.89 ± 0.04 ^a^	96.01 ± 0.20 ^a^	96.56 ± 0.10 ^a^
a*	0.21 ± 0.17 ^a^	0.46 ± 0.05 ^a^	−0.67 ± 0.09 ^b^
b*	1.25 ± 0.39 ^b^	3.44 ± 0.38 ^a^	3.49 ± 0.23 ^a^

L* is lightness, a* is the red/green coordinate, and b* is the yellow/blue coordinate. Different lowercase superscript letters in the same row indicate significant differences (*p* < 0.05).

**Table 3:** Functional properties of starch from three varieties of yam.

Functional Properties	*D. cayenensis*	*D. alata*	*D. rotundata*
Water absorption index, WAI (g/g)	2.39 ± 0.01 ^b^	2.46 ±0.02 ^c^	2.22 ± 0.01 ^a^
Water solubility index, WSI (%)	0.76 ± 0.05 ^a^	1.10 ± 0.10 ^b^	1.00 ± 0.10 ^b^
Swelling power, SP (g/g)	2.42 ± 0.04 ^b^	2.54 ± 0.06 ^c^	2.26 ± 0.04 ^a^

Different lowercase superscript letters in the same row indicate significant differences (*p* < 0.05).

**Table 4:** Gelatinization parameters of starch from three varieties of yam.

Variety of Yam	To (°C)	Tp (°C)	Tc (°C)	Tc-To (°C)	ΔH (J/g)
*D. rotundata*	75.16 ± 0.10 ^b^	79.63 ± 0.15 ^b^	85.64 ± 0.06 ^a^	10.48 ± 0.04 ^b^	4.19 ± 0.21 ^a^
*D. cayenensis*	74.02 ± 0.03 ^a^	79.11 ± 0.01 ^a^	85.72 ± 0.22 ^a^	11.70 ± 0.25 ^c^	4.30 ± 0.087 ^a^
*D. alata*	75.98 ± 0.11 ^c^	78.94 ± 0.25 ^a^	85.42 ± 0.30 ^a^	9.43 ± 0.42 ^a^	5.28 ± 0.10 ^b^

To, initial gelatinization temperature; Tp, maximum gelatinization temperature; Tc, gelatinization completion temperature; ΔH, gelatinization enthalpy. Different lowercase superscript letters in the same column indicate significant differences (*p* < 0.05).

**Table 5:** Pasting parameters of starch from three varieties of yam.

**Variety of Yam**	**PT (°C)**	**PV (cP)**	**FV (cP)**	**BD (cP)**	**SV (cP)**
*D. rotundata*	75.52 ± 0.10 ^a^	1634 ± 32 ^a^	2908 ± 54 ^a^	129 ± 21 ^a^	1403 ± 42 ^a^
*D. cayenensis*	73.62 ± 0.13 ^a^	1957 ± 28 ^b^	3538 ± 36 ^c^	150 ± 30 ^a^	1731 ± 55 ^c^
*D. alata*	74.40 ± 0.09 ^a^	2019 ± 41 ^b^	3057 ± 42 ^b^	465 ± 38 ^b^	1503 ± 53 ^b^

PT: pasting temperature; PV: peak viscosity; FV: final viscosity; BD: breakdown viscosity; SB: setback viscosity. Different lowercase superscript letters in the same column indicate significant differences (*p* < 0.05).

## Data Availability

The raw data supporting the conclusions of this article will be made available by the authors on request.
